# Optimising stakeholder engagement during intervention planning and development using the Person-Based Approach: the example of an online FeNO-guided asthma management intervention in primary care

**DOI:** 10.1038/s41533-025-00435-9

**Published:** 2025-07-25

**Authors:** Marta Santillo, Kate Morton, Michelle Helena Van Velthoven, Lucy Yardley, Mike Thomas, Kay Wang, Ben Ainsworth, Sarah Tonkin-Crine

**Affiliations:** 1 Nuffield Department of Primary Care Health Sciences, University of Oxford, Radcliffe Observatory Quarter, Woodstock Road, Oxford, UK; 2 Nuffield Department of Health Sciences, University of York, York, UK; 3 Centre for Clinical and Community Applications of Health Psychology, School of Psychology, Faculty of Environmental and Life Sciences, University of Southampton, Southampton, UK; 4 School of Psychological Science, University of Bristol, Bristol, UK; 5 Primary Care, Population Sciences and Medical Education (PPM), University of Southampton, Southampton, UK; 6 NIHR Health Protection Research Unit in Healthcare Associated Infections and Antimicrobial Resistance, University of Oxford, Oxford, UK

**Keywords:** Asthma, Health services

## Abstract

This paper is a detailed methodological analysis of how the PBA approach was used as part of the DEFINE programme, in the planning and development of a behavioural intervention to support the use of Fractional Exhaled Nitric Oxide (FeNO) in informing asthma management in primary care asthma reviews. It offers detailed research insights into how using the PBA approach facilitates the development of methodologies for stakeholder engagement and intervention development research, in line with the recent MRC framework. Two stakeholder workshops were organised during the intervention planning and development phases. The patient stakeholders were diverse in age, gender, and asthma severity, while the clinical stakeholders were diverse in clinical role and level of experience using FeNO. The research team mapped how the stakeholders’ feedback complemented the core research team-based activities during the two stages of intervention planning and development, and what the outcomes of such engagement were. The five PBA intervention development activities in which stakeholderswere involved were: (1) Understanding target behaviours; (2) Identifying how to promote engagement with target behaviours; (3) Ensuring anticipated mechanisms of action are taken into account in planning intervention components; (4) Developing intervention content; and (5) Identifying the best intervention content and implementation. Outcomes of involving stakeholder in the 5 intervention development activities were: in depth interpretation on the qualitative work,new barriers and facilitators to the target behaviour of adoption and use of FeNO test during asthma reviews, and optimisation of intervention materials through in-depth tailoring of the online training and patient leaflet.

## Introduction

Current standard methods of monitoring asthma in primary care mainly include patient-reported symptoms and basic lung function assessments. Pharmacologic therapy decisions, which could include the use of inhaled corticosteroids (ics) and dose adjustments, are frequently based on these self-reported assessments^1,2^. These methods are only weakly correlated to objective levels of airway inflammation, making them unreliable predictors of future exacerbations^3^.

In 2014, the UK National Institute for Health and Care Excellence (NICE) recommended that clinicians should use FeNO to diagnose asthma more accurately. These guidelines also recommended further research to assess the use of FeNO in monitoring of asthma^4^. The new joint BTS/NICE/SIGN guideline on asthma diagnosis and management advises that clinicians should consider FeNO monitoring at annual routine asthma reviews, but does not provide any specific guidance on how FeNO results should inform asthma management^5^. BTS/SIGN guidelines advise that clinicians should consider gradually reducing treatment in patients who have been clinically stable for three months or longer^6^. Knowing that a patient’s FeNO result is low would give clinicians more confidence that this will be safe for your patient. FeNO is a simple, non-invasive breath test that provides objective evidence of steroid-responsive eosinophilic airway inflammation. However, FeNO is not routinely measured in primary care, where the majority of asthma diagnosis and monitoring takes place^7^.

The DEFINE programme developed a behavioural intervention to include the FeNO test, FeNO test instructions, an online training module for clinicians, a patient information leaflet, and a FeNO-guided algorithm that produced personalised management recommendations for patients.

Stakeholder involvement throughout intervention planning and development is in line with best practice and helps ensure that an intervention is relevant, accurate, and compatible with local contexts^8^. Including varied stakeholder perspectives, and thereby reflecting all agents in a complex intervention, has also been identified as a priority by the recently updated MRC framework^9^. However, while case studies are provided by the MRC framework, the methodological detail they provide is variable. Therefore, an in-depth methodological analysis of how to incorporate stakeholder engagement throughout intervention planning and development should help to expand on this recommendation and reflect on the opportunities and challenges of such activities.

Stakeholder engagement is at the centre of the Person-Based Approach^10^ (PBA). PBA has been successfully used for the development of many digital interventions, including interventions for self-management of asthma^11–13^.

In order to maximise the research insights obtained, this paper provides a detailed methodological analysis of how stakeholder involvement was incorporated into PBA research activities throughout the planning and development of the DEFINE behavioural intervention. The qualitative research that complemented these development activities has already been reported previously in this journal^14^.

## The person-based approach

The main aim of the Person-Based Approach (PBA) is to gain detail undertstanding of the context of the people using the intervention through in-depth qualitative research^10^. PBA provides a process by which the intervention developer can gain detailed insight into how people experience and implement the intervention. This approach also provides a clear framework for developers to identify the key characteristics that will increase the engagement and utility of the intervention. However, there is often insufficient space in these papers to describe in full detail the stakeholder engagement during each of the research activities included in the intervention development process. This contributes to the lack of guidance on how to conduct stakeholder research^15^ and there is need for training and methods to support researchers to use stakeholders in their research projects^16^. PBA allows developers to understand how best to support the theory-based models and how different people will engage with different elements of the interventions in different contexts. Stakeholder and PPI input and in-depth qualitative research are central to the PBA^17,18^.

The PBA approach is also an iterative process. The key three stages of intervention development are intervention planning, intervention optimisation, and intervention implementation. Currently there are variations in how PBA implementation and research processes are reported in papers^17^. This paper focusses on the first two stages as these are the two stages conducted before the Trial started. In the first stage, intervention planning, published qualitative and mixed methods work can be used to identify barriers and facilitators and contextual issues relevant to the target behaviours of the intervention^19^. Insights from published work and in-depth qualitative interviews inform the design of the guiding principles which specify the design objectives of the intervention, and features of the intervention which will achieve these objectives. During the second stage, intervention optimisation, in-depth interview techniques, such as ‘think-aloud’ interviews, are used to optimise interventions^20^. They allow designers to understand how members of the target population would use the intervention. Changes to the interventions are made iteratively and this allows further rounds of think-aloud interviews.

Stakeholder (which includes clinicians and patient representatives) involvement and in-depth qualitative research are central to the PBA, informing intervention planning, and also subsequent phases such as intervention development, evaluation and implementation. Involving stakeholders in this way meant that they effectively co-developed the intervention with us, making is more likely that the intervention would be feasible and acceptable to them, and address issues which they felt were important.

In the DEFINE programme, the research team recorded and mapped stakeholder contributions at each stage of intervention planning and development through researchers’ notes and summarising of these in a planning table. We collated these to understand how stakeholders helped to optimise the intervention.

## The define programme

### Aims

The DEFINE (Development and Evaluation of an online FeNO-guided asthma management INtervEntion in primary care) programme aimed to develop a behavioural intervention package to support the use of Fractional Exhaled Nitric Oxide (FeNO) in informing asthma management in primary care asthma reviews.

The DEFINE intervention was developed following principles of the PBA and included an enhanced in depth series of stakeholder engagement activities which accompanied the activities of the core research team at each stage of the intervention development. The aim of using the PBA and stakeholder engagement was to make sure that the intervention would be engaging and useful for patients and clinicians.

The online training supported clinicians to use the FeNO test, how to explain to patients how to do the test, how to interpret a FeNO result using the algorithm, and how to explain the algorithm recommendations to patients. A patient leaflet was provided to explain how to do the test and how their result could help inform more personalised management of their asthma. Table 1 provides the description for each intervention material.

**Table 1:** DEFINE intervention materials.

Intervention materials	Description
**For clinicians**	
Online training	An online training module on what FeNO is and how to use the FeNO algorithm during asthma reviews, which includes patient scenarios, video consultations and champions’ testimonies, to increase clinicians’ confidence and belief in using FeNO to provide more personalised asthma management.
FeNO algorithm	An online algorithm that offers personalised recommendations on asthma management based on ACT score, FeNO test result, number of asthma exacerbations in the last 12 months, and other information about the patient’s asthma and general health to support clinicians to use a FeNO test result to inform management decisions. Subsequent questions in the algorithm after the 3 initial pieces of information are tailored according to the answers which clinicians give to the questions which are presented to them.
FeNO test instructions	The instructions are double sided. One side explains to clinicians how to set up the machine and what the various components and screen icons are and the other is a step by step guide for patients on how to actually do the test.
**For patients**	
Information leaflet entitled “Your Asthma Review – How FeNO can help”	A leaflet to inform patients about FeNO, how to do a test and what a FeNO test result means, to increase their belief that they will be able to carry out the FeNO test, and that FeNO supports more personalised asthma management which is beneficial to them.

## Stakeholder involvement methods

Based on professional experience and guidance on best practice, the stakeholder engagement plan was developed. The core research team agreed on how many workshops and consultations to organise. Format, length and other practical details were agreed based on the specific stage of the intervention development. We planned for the stakeholder engagement to follow the PBA principles. This meant that we included stakeholders input at all stages of the development of the DEFINE programme. Initial design was informed by comments and suggestions from stakeholders (we had not designed the intervention yet at the time of the first stakeholder meeting).We agreed on the number but the content depended on the stage of the intervention development and it was iterative as we often came back to revise the intervention plan and materials as neededStakeholder involvement was included to all key decisions about intervention planning and optimization rather than just feedback to activities completed by the core research team.

The research team organised two workshops with stakeholders during the intervention planning and development process. The first workshop was hybrid (two stakeholders attended in person, the rest were online). However, due to the COVID-19 pandemic we were subsequently unable to engage in face-to-face contact with stakeholders, so we communicated regularly via email and held the second workshop remotely. Stakeholders were consulted by email and by conference calls at several points between and after the two workshops. Table 2 provides a summary of the participants, and the aims and outcomes of the two workshops.

**Table 2:** Summary of Stakeholder workshops.

Workshop	Stakeholder Participants	Aims	Feedback	Outcome
Workshop 1 (face to face and remote, March 2020)	2 PPI contributors (one adult, one young person), 1 clinical pharmacist, 1 senior nurse practitioner, 1 GP, 1 Consultant Paediatric Respiratory Physician, 2 Respiratory Medicine Professors and 1 Professor of Primary Care Research	To elicit views on intervention components	• how to present the content of the web-based tool • what to include in the online training and how to best design the case scenarios • what questions to address in the patient leaflet	Identification of additional barriers and facilitators to the use of the FeNO test and algorithm during asthma reviews reported in the behaviour analysis table Development of initial intervention components and materials (contribution to clinical content of online training, patient leaflet and FeNO algorithm reccomendations)
Workshop 2 (remote, October 2020)	2 PPI contributors (two adults), 1 Clinical Pharmacist, 1 Consultant Paediatric Respiratory Physician, 1 GP, 2 Professors of Primary Care Research	To elicit feedback on intervention components and guiding principles To elicit views on implementation of algorithm and FeNO test	• how to use the online training to support clinicians to conduct remote consultations and safely conduct face-to-face reviews during the Covid-19 pandemic • how to store the FeNO machine in practice • how to present the leaflet to ensure that patients who feel they have well controlled asthma can also relate to it • how to make the patient leaflet more convincing and attractive	Refinement of clinical content of digital materials (online training and algorithm, patient leaflet)

In order to analyse the stakeholder feedback members of the research team audio recorded the discussions, took detailed notes, and provided a written summary of the discussions and key comments emerging from the discussions. The written feedback was shared between the core research team and the stakeholder group. The core research team used the summaries to complete any research output from each PBA intervention development activity (behavioural analysis table, table of changes). Any changes to intervention materials and documents as a result of stakeholder discussions were shown to stakeholders for further feedback and agreement. Written consent was not required for the workshop as these were PPIE activities. Researchers obtained verbal consent for the recordings.

### Stakeholder panel

A stakeholder is anyone who has a stake in the intervention, including potential users and prioviders. In the specific case of the DEFINE intervention we decided to include both patients and clinicians who would conduct primary care asthma reviews. Stakeholders were an external group of collaborators who were either clinical or patient representatives. Stakeholders were independent from the research team and were acting in an advisory capacity only in relation to introduction of the intervention in primary care - they did not have any input into the management or delivery of the research. Among the clinicians we had stakeholders who knew about the test in both primary and secondary care, and who would conduct regular asthma reviews in primary care. Among the patients we had patients with asthma who were not familiar with FeNO. The Core research team was a group of academics who developed and run the DEFINE programme. They had a research background in behaviour science in primary care and academic GPs with expertise in respiratory care. We aimed to recruit diverse stakeholders from these two groups. The patient stakeholders were diverse in age, gender, and asthma severity. Clinicians stakeholders were diverse in clinical role and experience of using FeNO during asthma reviews. Among the clinician stakeholder we included 1 clinical pharmacist, 1 senior nurse practitioner (advance practice registered nurse), 1 GP, 1 Consultant Paediatric Respiratory Physician, 2 Respiratory Medicine Professors and 1 Professor of Primary Care Research.

Clinicians and Patient and Public Involvement (PPI) contributors were identified from Asthma UK, personal networks, existing asthma groups, such as Asthma UK and groups of children/young people with asthma and snowballing. In the manuscript we will refer to the clinicians and patients groups together under the term of “stakeholder group” as they both conducted the same PBA activities for the project. In case of different contributions or opinions we will specify which subgroup we will refer to.

## PBA intervention development activities

**Fig. 1:**
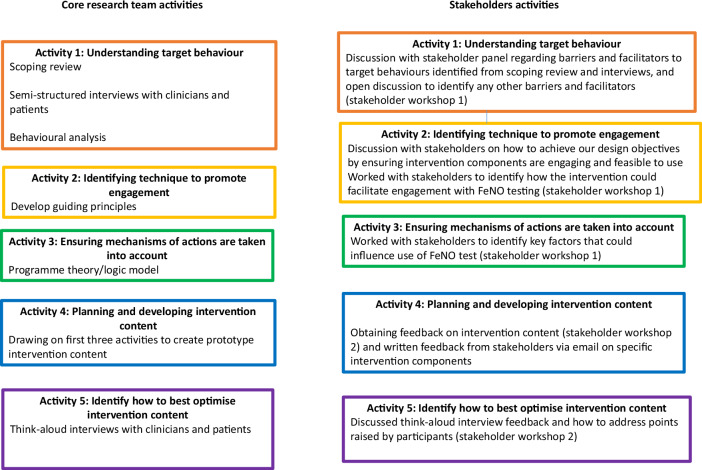
Visual representation of the 5 PBA intervention development activities.

The research team and the stakeholder activities – i.e. provision of input and feedback into the process – are presented below and summarised in Table 3. As Table 3 shows each workshop covered multiple PBA intervention development activities. The core research work and stakeholder work for each activity occurred either separately or simultaneously depending on the specific activity. The intervention development in each activity was an iterative process so oftern activities concurred at the same time, rather than one after the other. Figure 1 includes a visual representation of the 5 PBA intervention development activities.

**Table 3:** Research team and stakeholder activities contributing to intervention planning and development.

PBA Activities	Research team activity	Stakeholder activity
1. Understanding target behaviours	Scoping review Semi-structured interviews with clinicians and patients Behavioural analysis	Discussion with stakeholder panel regarding barriers and facilitators to target behaviours identified from scoping review and interviews, and open discussion to identify any other barriers and facilitators (stakeholder workshop 1)
2. Identifying techniques for promoting engagement with target behaviours	Develop guiding principles	Discussion with stakeholders on how to achieve our design objectives by ensuring intervention components are engaging and feasible to use Worked with stakeholders to identify how the intervention could facilitate engagement with FeNO testing (stakeholder workshop 1)
3. Ensuring anticipated mechanisms of action are taken into account in planning intervention components	Programme theory/logic model	Worked with stakeholders to identify key factors that could influence use of FeNO test (stakeholder workshop 1)
4. Planning and developing early intervention content	Drawing on first three activities to create prototype intervention content	Obtaining feedback on intervention content (stakeholder workshop 2) and written feedback from stakeholders via email on specific intervention components
5. Identifying how best to optimise the intervention content and delivery	Think-aloud interviews with clinicians and patients	Discussed think-aloud interview feedback and how to address points raised by participants (stakeholder workshop 2)

### Activity 1: Understanding target behaviours

#### Core research team

The research team included the researchers who developed the DEFINE program. They conducted semi-structured interviews with 23 clinicians and 22 patients to explore their views on asthma reviews and asthma management and identify barriers and facilitators to the adoption and use of FeNO in primary care asthma reviews; the specific methods and results of this research activity have been published previously^14^. The findings highlighted how both patients and clinicians would value FeNO as an objective measure to educate patients about airway inflammation and to support clinicians with providing a more personalised asthma management plan. The research team also undertook a behavioural analysis to understand target behaviours and map these onto known theoretical constructs^21^ (see table in Additional File 1).

#### Stakeholders

Stakeholder discussions led to an in-depth understanding of the target behaviours for patients and clinicians. Target behaviours for patients were: to complete the FeNO test during a consultation; and to follow the asthma management plan. Clinicians’ target behaviours were: to explain the FeNO test to patients; conduct the FeNO test; use the FeNO-guided algorithm; follow the algorithm recommendations; and to discuss the management plan with patients. From the discussion, the research team extrapolated a list of barriers and facilitators from both clinicians and patients in the stakeholder group which were added to the list of the ones identified through the scoping review and qualitative study. For example, clinicians and PPI representatives highlighted the importance of providing clear and credible information about FeNO testing to patients both before and during the asthma review, to help them see the benefits of this procedure, and to increase engagement with the asthma management recommendations resulting from the asthma review. The full behavioural analysis is included in Additional file 1.

### Activity 2: Identifying how to promote engagement with target behaviours

#### Core research Team

In line with the PBA approach^10^, guiding principles were used to highlight how interventions address key issues that are crucial to engaging users. The guiding principles were developed by the research team based on the evidence collated during Activity 1, and then refined throughout the intervention development process.

#### Stakeholders

At this stage, stakeholders suggested ways for the intervention to promote clinician’s engagement with the algorithm. They did so at multiple time point, making their feedback part of the iterative process. They advised using simpler language and preferences regarding the layout for the algorithm. They also suggested adding clinical scenarios to the clinician online training.

To increase users’ engagement with the intervention materials, clinicians from the stakeholder groups supported the research team in designing materials including a video of how to do a FeNO test which could help inform and reassure patients. The research team also produced two video consultations of mock consultations which clinician stakeholders helped script and produce. The consultations represented clinical scenarios which the research team felt might be quite common in primary care and which clinicians could potentially find difficult to navigate if they were unfamiliar with using FeNO to guide clinical decision-making during asthma reviews.

Stakeholders suggested that the online training could be designed – both in terms of look and navigation – to mirror existing clinician training modules, so that clinicians would be familiar with the format. These changes are summarised in Tables 2 and 3.

### Activity 3: Ensuring anticipated mechanisms of action are taken into account in planning intervention components

#### Core research team

The research team created a logic model (Additional file 2) as a visual representation of the hypothesised processes and causal pathways by which the intervention would lead to the desired outcomes^22^. The logic model was the reviewed and refined at later stages of the intervention development.

The research team theorised that the intervention would work by increasing clinicians’ confidence in incorporating FeNO into asthma reviews, and increasing their belief about positive consequences of changing patients’ management based on FeNO readings. Its effects would be achieved through changes to clinicians’ cognitions and behaviours, and to a lesser extent the intervention would also likely change patients’ beliefs about their asthma and their behaviours. Specifically, having an objective measure of inflammation was theorised to result in improved adherence to medication in patients with low adherence through changing their perceived need for the medication.

#### Stakeholders

The research team discussed mechanisms of actions with the stakeholder group. One of the main concerns from the stakeholders was whether clinicians would be willing to step down asthma medication when recommended by the algorithm due to a low FeNO result. Stakeholders underlined that the aim would be to get clinicians to *consider* stepping down even if they do not actually step down medication. They highlighted that there can be valid reasons for not stepping down a patient’s medication at a particular time (e.g. being about to enter the hay fever season when hay fever is a known trigger for the patient’s asthma symptoms). The decision should be based on the whole clinical picture, not just the FeNO result/algorithm recommendation.

Stakeholders reflected that patients with well controlled asthma may feel nervous about stepping down treatment and patients in the group suggested ways in which the patient leaflet may be used to empower patients in feeling more comfortable and safe with a decision of stepping down medication.

Discussions with stakeholders indicated that few teams in general practice had previously used FeNO analysers, but in general were very keen to have the opportunity to incorporate FeNO testing into asthma reviews via this study, due to the perceived benefit of the additional clinical information and the opportunity to trial a FeNO test for free.

The stakeholders provided suggestions for the use of the FeNO-guided algorithm in routine reviews whilst considering some concerns – for example, the risk of changing the dynamics of the consultation if the clinician is looking mainly at a computer screen rather than the patient, the length of consultations, and that the test and algorithm would be mainly used by nurses. This is because most routine asthma reviews in primary care are done by nurses.

Discussions with stakeholders informed the decision to define adherence in the trial as clinicians showing evidence that the recommendations of the algorithm were considered and discussed with the patients.

### Activity 4: Develop intervention content

#### Core research team

Based on activities 1–3 the research team designed the first version of the intervention components. Stakeholders were asked for their feedback on the initial intervention materials and then specifically asked to comment on their views of clinician adherence to the algorithm that was highlighted as the key target behaviour for clinicians.

#### Stakeholders

Clinicians were concerned about the length and wording of the algorithm recommendations and suggested opting for simpler language, in order to break down information for busy clinicians during asthma reviews and to facilitate communication of the management plan to patients.

They also agreed on the importance of introducing clinical scenarios to facilitate the understanding of particular clinical issues with which FeNO could help. The case scenarios were designed by both stakeholders and researchers to provide examples of how to communicate with patients and address clinical scenarios that were common scenarios which would be encountered and managed in primary care, such as stepping down asthma medication. These changes are summarised in Tables 2 and 3.

### Activity 5: Identifying how best to optimise the intervention content and implementation

#### Core research team

During the optimisation phase of the intervention materials, the research team conducted think-aloud interviews with 11 clinicians and 7 patients to hear their thoughts on intervention materials. The methods and results of the think-aloud interviews are reported elsewhere^23^. We have provided more information on the output of the think-aloud interviews and included them as supplementary materials S3.

As part of the think-aloud process the core research team produced a table of changes which listed all comments made by clinicians and patients interviewed. Possible changes and priorities were identified, made and then presented and final consensus was reached including the stakeholder feedback. An extract from the table of changes is presented in supplementary material 3 (S3). The outcome of the interviews was the iterative development of the final intervention materials.

#### Stakeholders

The results of these think-aloud interviews were discussed in stakeholder workshop 2. Stakeholders suggested some changes to the intervention materials based on the interview feedback. Clinicians suggested refinements to the content and wording of the algorithm and online training, such as how to emphasise in the online training that a high or intermediate FeNO result required further exploration even if the patient’s asthma symptoms appeared to be well managed. Stakeholders also suggested adding new content to the online training regarding how to use the FeNO test in both face-to-face and remote asthma reviews. Stakeholders reflected that patients with well-controlled asthma may feel nervous about stepping down treatment and content to address this needed to be added to the patient leaflet. These changes are summarised in Tables 2 and 3.

## Discussion

This paper provides a detailed analysis of the methodology used to optimise stakeholder engagement in the planning and development of an online FeNO-guided asthma management intervention. It presents an in-depth analysis of how the Person-Based Approach (PBA) was followed, which included regular contact input from stakeholders – including clinicians and PPI contributors – at all five stages of the development, and optimisation of intervention components and materials.

### Lesson learnt through stakeholder engagement

This degree of stakeholder engagement helped to identify additional barriers and facilitators to the use of the FeNO test and associated algorithm for use in primary care compared to the ones identified from a literature review and the qualitative interviews^14^. It also supported the explanation of the formal identification of the problem, outcomes of the interventions, and how the intervention works.

They also informed decisions about how best to develop and optimise the four key intervention components. PPI representatives supported the tailoring of the key messages in the patient leaflet to patients with well controlled or poorly controlled asthma, and provided useful insight on how to make the leaflet more accessible and concise to patients.

Clinicians highlighted the importance of including positive examples of how to explain the FeNO test to patients, and of structuring the online training around clinical scenarios of patients with different levels of asthma control and FeNO test results.

### Strengths and limitations

A strength of this PBA-based research methodology was the varied stakeholders involved in the workshops and consultations, bringing together various expertise and patients and clinicians, throughout multiple stages of the intervention planning and development process. This made the process of gaining feedback on new ideas on such materials during the intervention planning more efficient. Although some of the clinicians in our stakeholder group had an academic background, we involved other clinicians with only clinical roles such as nurse practitioner and clinical pharmacists.We would have preferred further representation from some groups of clinicians such as more non-academic GPs and more pharmacists and nurses practitioners. The clinical pharmacist in the stakeholder group did try to find more pharmacists but did not get a positive response. Although we had only one nurse practitioner who was officially a member of the stakeholder group, we did have 4 additional nurse practitioners who contributed and helped us in the development of the FeNO algorithm clinical content. The patient stakeholders were diverse in age, gender, and asthma control, although recruitment was not able to address all other relevant demographic factors (e.g. digital literacy, social deprivation indices, and ethnicity).

We have added a paragraph in the discussion around challenges and our experiences of managing such challenges including disagreement.

It is also important to discuss how to deal with potential challenges when combining research and stakeholder activities. We believe that the iterative and person-centered nature of the PBA helped us to address any disagreements and challenges. We valued any contrasting opinions that may have risen from the stakeholder groups.

For the research team it was important taking what each group/person says and checking that view with a wider group. The different inputs complement each other and expand our understanding of what needs to be addressed in the intervention.

Stakeholder engagement is increasingly recognised as a crucial aspect of intervention development, and recent work on intervention planning has been developed with a focus on how to include stakeholder engagement and qualitative research at the heart of PBA. Recent iterations of the Person-Based Approach have been updated to more explicitly integrate stakeholder feedback alongside qualitative research and it is vital to ensure that co-participatory approaches to including stakeholder feedback are reviewed and updated to ensure best-practice^17^. Stakeholder engagement has previously been used as part of the PBA approach to development of digital interventions in secondary care^21^. However, a recent review of stakeholder engagement in behaviour change research^24^ highlighted that the evidence of the impact of stakeholder engagement on behaviour change intervention research is still underdeveloped^25^.

## Conclusions

This paper provides a detailed observational analysis of how stakeholders were incorporated (PBA) which illustrates a methodological framework, suggesting that theory can be translated into practice. It describes how stakeholder engagement could be used in alignment with research activities in the development of behavioural interventions, and how this benefited the development of intervention components and materials so they could be better tailored to target groups. Stakeholder workshops and consultations are part of the iterative process and can mirror the five key PBA activities included in the stages of intervention development.

## Supplementary information

S1

S2

S3

## Data Availability

No datasets were generated or analysed during the current study.
