# Supplementary material for: Optimising stakeholder engagement during intervention planning and development using the Person-Based Approach: the example of an online FeNO-guided asthma management intervention in primary care
Source: NPJ Prim Care Respir Med. 2025 Jul 25;35:33. doi: 10.1038/s41533-025-00435-9 (PMC12297380; doi:10.1038/s41533-025-00435-9)
Supplement: Supplementary file 1 — S1 [file 41533_2025_435_MOESM1_ESM.pdf]

| **Barriers / *facilitators* to target behaviours** | **Intervention Component/s** | **Intervention ingredient** | **Theoretical Domains Framework (TDF)** | **Target construct (BCW)** | **Intervention function (BCW)** | **BCT (using 93 BCT taxonomy v1)** |
|---|---|---|---|---|---|---|
| **Patient: Complete the FeNO test during consultation** | | | | | | |
| *Patients want to know what FeNO does, and what the results mean (EC)* | Patient leaflet<br><br>Discussion about FeNO measurement and test | Provide written information in advance to explain the purpose of FeNO testing and how it can inform asthma management<br><br>Provide support during consultation to ensure patient understands the purpose of FeNO testing | Knowledge | Psychological capability | Education | 5.1 Information about health consequences<br><br>5.2 Salience of consequences<br><br>9.1 Credible source |
| *Patients want to know what the benefits for them are (EC)* | Patient leaflet<br><br>Discussion about FeNO measurement and test | Provide written information in advance to explain how FeNO testing can better inform asthma management<br><br>Provide support during consultation to explain how FeNO testing can better inform asthma management | Knowledge<br><br>Beliefs about consequences | Psychological capability<br><br>Reflective motivation | Education<br><br>Persuasion | 5.1 Information about health consequences<br><br>5.2 Salience of consequences<br><br>9.1 Credible source |

| **Barriers / *facilitators* to target behaviours** | **Intervention Component/s** | **Intervention ingredient** | **Theoretical Domains Framework (TDF)** | **Target construct (BCW)** | **Intervention function (BCW)** | **BCT (using 93 BCT taxonomy v1)** |
|---|---|---|---|---|---|---|
| *Patients want to know how they have to breathe in (EC)*<br><br>Doesn't think she could breathe out for 10 seconds, she always have a cough when she tried (I, p0109)<br><br>Some patients may struggle blowing for 10 seconds (I, p0111)<br><br>Not sure how hard it is to breathe in the FeNO machine (I, p0112) | Patient leaflet and video<br><br>Discussion about FeNO measurement and test | Provide written and visual information in advance to explain how to take a FeNO test<br><br>Provide written and visual information on a handout for patient to use during consultation, with verbal explanation from HCP | Skills | Physical capability | Training | 4.1 Instruction on how to perform the behaviour<br><br>6.1 Demonstration of the behaviour |
| *Patients want to know If the FeNO test hurts (EC)* | Patient leaflet and video<br><br>Discussion about FeNO measurement and test | Provide written and visual information about the FeNO test being safe and easy | Emotion | Automatic motivation | Persuasion | 11.2 Reduce negative emotions |

| **Barriers / *facilitators* to target behaviours** | **Intervention Component/s** | **Intervention ingredient** | **Theoretical Domains Framework (TDF)** | **Target construct (BCW)** | **Intervention function (BCW)** | **BCT (using 93 BCT taxonomy v1)** |
|---|---|---|---|---|---|---|
| *The patient information needs to be clear but not patronising (EC)*<br><br>*Leaflet needs pictures (EC)* | Patient leaflet<br><br>Discussion about FeNO measurement and test | Provide information that is accessible and visual | Memory, attention and decision process | Psychological capability | Education | 5.2 Salience of consequences |
| *Patients have positive views of using tests like FeNO, because they are an objective measure (EC)*<br><br>*It would be good to have quantitative feedback about asthma (I, p0110)*<br><br>*FeNO tests would give clinicians the right information (I, p0113)*<br><br>*They [patients] don't like it when it seems like a tick box exercise (I, HCP 0209)* | Patient leaflet<br><br>Discussion about FeNO measurement and test | Explain that FeNO gives objective information on inflammation, which can differ from how you feel, to highlight the benefit of FeNO testing | Beliefs about consequences | Reflective motivation | Persuasion | 5.1 Information about health consequences<br><br>5.2 Salience of consequences |
| **Patients: To agree to the asthma management plan** | | | | | | |

| **Barriers / *facilitators* to target behaviours** | **Intervention Component/s** | **Intervention ingredient** | **Theoretical Domains Framework (TDF)** | **Target construct (BCW)** | **Intervention function (BCW)** | **BCT (using 93 BCT taxonomy v1)** |
|---|---|---|---|---|---|---|
| *Patients want to know how the FeNO test informs the decision process (EC)* | Patient leaflet<br><br>Discussion re management plan<br><br>Written plan for patient to take away | Provide written information about the meaning of possible FeNO test results and the implications for asthma management<br><br>Discuss the personalised recommendations with the patient during the consultation<br><br>Introduced after Wave 1 of feasibility study: Written copy of recommendations based on FeNO and other factors | Knowledge<br><br>Beliefs about consequences | Psychological capability<br><br>Reflective motivation | Education<br><br>Persuasion | 5.1 Information about health consequences<br><br>9.1 Credible source |

| **Barriers / *facilitators* to target behaviours** | **Intervention Component/s** | **Intervention ingredient** | **Theoretical Domains Framework (TDF)** | **Target construct (BCW)** | **Intervention function (BCW)** | **BCT (using 93 BCT taxonomy v1)** |
|---|---|---|---|---|---|---|
| Reviews seem superfluous because patient knows that nothing needs to change (I, p0110, p109) | Patient leaflet | Provide written information about the additional information FeNO can provide about asthma management, to help patients see the benefit of the review when FeNO testing is added | Beliefs about consequences | Reflective motivation | Persuasion | 5.1 Information about health consequences |
| Harsh cut off points between low, intermediate, high FeNO (I, p0111) | Discussion re management plan | Discuss the personalised recommendations with the patient during the consultation so they can see how their FeNO score and other factors have influenced their management plan | Beliefs about consequences | Reflective motivation | Persuasion | 9.1 Credible source |
| **Patient: To carry out behaviours advised in the asthma management plan** | | | | | | |

| **Barriers / *facilitators* to target behaviours** | **Intervention Component/s** | **Intervention ingredient** | **Theoretical Domains Framework (TDF)** | **Target construct (BCW)** | **Intervention function (BCW)** | **BCT (using 93 BCT taxonomy v1)** |
|---|---|---|---|---|---|---|
| Young people lack confidence and skills in communicating their health concerns and asking questions (LR) | Discussion about FeNO measurement and test<br><br>Discussion about management plan | HCP will invite questions about the FeNO test beforehand, and about the management plan afterwards, to try and create a safe and comfortable environment to communicate concerns or questions | Social influences | Social opportunity | Environmental restructuring | 12.2 Restructuring the social environment |
| *Pre-consultation guide promoted self-efficacy and increased confidence in asking questions (LR)* | Patient leaflet | Promote the opportunity for shared decision making to decide how to incorporate FeNO test results into a personalised asthma management plan | Beliefs about capabilities | Reflective motivation | Persuasion | 3.1 Social support |

| | | | | | | |
|---|---|---|---|---|---|---|
| *Patients' values and preferences are considered* (LR)<br><br>*Patients want to feel involved, being proactive (EC)*<br><br>Patients' preferences are often assumed, not elicited (LR)<br><br>*Increased patient information, autonomy, and control over treatment decision* (LR)<br><br>Lack of direct involvement during consultations limits opportunities for young people to learn to self-manage and gain better control of their asthma (LR)<br><br>Patients would like to be more involved in treatment decisions (LR) | Discussion about management plan<br><br>HCP online training<br><br>Patient leaflet | HCP will invite patients to share their preferences for asthma management<br><br>HCP training asks HCPs to consider patients' preferences when making an asthma management plan, e.g. "If your patient is happy to try reducing their medication, the FeNO web tool recommends that you re-check their FeNO in 6 to 8 weeks' time"<br><br>Patient-HCP videos of Shakila and Peter model HCPs having discussion with patient which incorporates their preferences and understanding: e.g. "I completely understand your concern with that Peter" | Social influences<br><br>Skills<br><br>Beliefs about consequences | Social opportunity<br><br>Psychological capability<br><br>Reflective motivation | Environmental restructuring<br><br>Training<br><br>Persuasion | 12.2 Restructuring the social environment<br><br>4.1 Instruction on how to perform the behaviour<br><br>9.1 Credible source<br><br>5.1 Information about health consequences<br><br>1.4 Action planning |

| **Barriers / *facilitators* to target behaviours** | **Intervention Component/s** | **Intervention ingredient** | **Theoretical Domains Framework (TDF)** | **Target construct (BCW)** | **Intervention function (BCW)** | **BCT (using 93 BCT taxonomy v1)** |
|---|---|---|---|---|---|---|
| *Decrease of the power asymmetry between HCPs and patients* (LR)<br><br>*Giving patients ownership would improve patient adherence to medication* (LR)<br><br>*Patients like to be engaged in the decision making process, to feel they are important, they are part of it (I, HCP 07)* | | Patient leaflet emphasises that their preferences will be taken into account in deciding an action plan, e.g. "It may be possible to safely lower the dose of your medication. If you feel this is right for you, talk to your doctor or nurse" | | | | |

| **Barriers / *facilitators* to target behaviours** | **Intervention Component/s** | **Intervention ingredient** | **Theoretical Domains Framework (TDF)** | **Target construct (BCW)** | **Intervention function (BCW)** | **BCT (using 93 BCT taxonomy v1)** |
|---|---|---|---|---|---|---|
| Children lack knowledge about asthma (LR)<br><br>*Patients want to understand their condition to better control it (EC)* | Patient leaflet<br><br>Discussion about FeNO measurement and test<br><br>Discussion about management plan | Provide explanation in clear language about what the FeNO test measures and what it means about your asthma<br><br>HCP will explain about FeNO testing and possible implications for managing asthma | Knowledge | Psychological capability | Education | 5.1 Information about health consequences |
| *It encourages them to use the steroids, they have a tangible number (I, pharmacist 07)* | Discussion about management plan | Provide explanation in clear language about what the FeNO test measures and what the result means in terms of medication | Knowledge | Psychological capability | Education | 5.1 Information about health consequences |
| **HCP: To explain FeNO measurement and test to patient** | | | | | | |

| **Barriers / *facilitators* to target behaviours** | **Intervention Component/s** | **Intervention ingredient** | **Theoretical Domains Framework (TDF)** | **Target construct (BCW)** | **Intervention function (BCW)** | **BCT (using 93 BCT taxonomy v1)** |
|---|---|---|---|---|---|---|
| *Positive examples of how they would explain FeNO during consultation (EC)* | Online training: videos | One patient-HCP video during session 1 of training which shows how to explain FeNO to a patient and how to take a FeNO reading<br><br>Four patient-HCP videos at the end of session 2 which model how to interpret FeNO results with a patient | Skills | Psychological capability | Training | 4.1 Instruction on how to perform the behaviour<br><br>9.1 Credible source |
| **HCP: Conduct the FeNO test** | | | | | | |
| Cost (LR)<br><br>Concern could be the cost (I, HCP 0208) | FeNO monitor | Provide FeNO monitor to Practices | Environmental Context and Resources | Physical opportunity | Environmental restructuring | 12.5 Adding objects to the environment |

| | | | | | | |
|---|---|---|---|---|---|---|
| *FeNO could be a great tool to assess adherence (I, Pharmacist 08)*<br><br>*Could help to understand why they are not responding to corticosteroids (I, Pharmacist 08)*<br><br>*it is useful to have a tangible objective number, that shows inflammation and response to steroids (I, Practice nurse 09)*<br><br>*Could support diagnosis and treatment for intermediate scores (I, Nurse 10)*<br><br>*Patients may think their asthma is good but then they are getting some symptoms (I, Nurse 10)* | Online training | Clear explanation of what FeNO measures, how this contributes to asthma management, and case study scenarios showing how specific FeNO scores contributed to asthma management decisions | Beliefs about consequences | Reflective motivation | Persuasion | 5.1 Information about health consequences |

| **Barriers / *facilitators* to target behaviours** | **Intervention Component/s** | **Intervention ingredient** | **Theoretical Domains Framework (TDF)** | **Target construct (BCW)** | **Intervention function (BCW)** | **BCT (using 93 BCT taxonomy v1)** |
|---|---|---|---|---|---|---|
| Concern is the accuracy of the test, would like to know more about what to do in the case of false results (I, Pharmacist 08)<br><br>Sensitivity and specificity (I, GP 11) | Online training | Explain that the FeNO analyser will not provide a reading unless the patient's technique was valid | Beliefs about consequences | Reflective motivation | Education<br>Persuasion | 9.1 Credible source |
| Practices are unsure because NICE guidelines say it is gold standard but BTS say it is a nice thing if you need it (I, Practice nurse 09) | Online training | Explain how FeNO fits with national guidance for asthma management | Social/Professional Role and Identity | Reflective motivation | Education | 3.1 Social support |

| **Barriers / *facilitators* to target behaviours** | **Intervention Component/s** | **Intervention ingredient** | **Theoretical Domains Framework (TDF)** | **Target construct (BCW)** | **Intervention function (BCW)** | **BCT (using 93 BCT taxonomy v1)** |
|---|---|---|---|---|---|---|
| *It is a simple and quick test (I, Pharmacist 07)*<br><br>*The FeNO test looks so simple and quick (I, Nurse 10)* | Online training<br><br>FeNO test<br><br>FeNO handout | Explain how to administer FeNO test and encourage HCP to try it themselves to reassure them that it is straightforward to administer.<br><br>Provide a short handout that HCPs and patients can use during the review | Skills<br><br>Memory, attention, decision processes | Psychological capability<br><br>Psychological capability | Training<br><br>Training | 4.1 Instruction on how to perform the behaviour<br><br>7.1 Prompts/cues |
| Would like to know if it is aerosol generating (I, Nurse 10) | Online training | Reassure HCPs that FeNO testing is not aerosol generating. | Knowledge | Psychological capability | Education | 5.1 Information about health consequences |
| Would like to know how to incorporate it in the template system (I, Nurse 10) | Online training | Explain how this fits with a routine asthma review | Skills | Psychological capability | Training | 4.1 Instruction on how to perform the behaviour |
| **HCP: Use the FeNO web tool** | | | | | | |

| **Barriers / *facilitators* to target behaviours** | **Intervention Component/s** | **Intervention ingredient** | **Theoretical Domains Framework (TDF)** | **Target construct (BCW)** | **Intervention function (BCW)** | **BCT (using 93 BCT taxonomy v1)** |
|---|---|---|---|---|---|---|
| If web based, they only want to see what they need *(EC)* | FeNO Web Tool | Web tool algorithm operates in the background and HCPs will only see questions and advice tailored to the information they have entered | Environmental Context and Resources | Physical opportunity | Environmental restructuring | 12.1 Restructuring physical environment |
| Risk of changing dynamic of consultation from discussion to just looking at screen *(EC)* | FeNO Web Tool<br><br>Discussion about management plan | Provide a web tool which is rapid to use, and does not involve much reading during consultation<br><br>Include interactive questions to ask the patient to facilitate a dialogue and show how the recommendations are informed by the patient's situation | Environmental Context and Resources<br><br>Social influences | Physical opportunity<br><br>Social opportunity | Environmental restructuring<br><br>Environmental restructuring | 12.1 Restructuring physical environment<br><br>12.2 Restructuring the social environment |

| **Barriers / *facilitators* to target behaviours** | **Intervention Component/s** | **Intervention ingredient** | **Theoretical Domains Framework (TDF)** | **Target construct (BCW)** | **Intervention function (BCW)** | **BCT (using 93 BCT taxonomy v1)** |
|---|---|---|---|---|---|---|
| *Information on how to use the FeNO web tool, how to interpret results (EC)* | Online training | Provide short user videos of how to input data to the FeNO web tool and show how it generates personalised recommendations<br><br>Provide 4 patient scenarios demonstrating how to interpret different FeNO results | Skills | Psychological capability | Training | 4.1 Instruction on how to perform the behaviour<br><br>9.1 Credible source |
| *Sometimes the FeNO score did not make sense (I, HCP 07)* | FeNO web tool | Use of a clinical algorithm to provide personalised recommendations for patients | Memory, Attention and Decision Processes | Psychological capability | Enablement | 7.1 Prompts/cues |

| Barriers / ***facilitators*** to target behaviours | Intervention Component/s | Intervention ingredient | Theoretical Domains Framework (TDF) | Target construct (BCW) | Intervention function (BCW) | BCT (using 93 BCT taxonomy v1) |
|---|---|---|---|---|---|---|
| *Visual aids* (LR) | FeNO Web Tool | Include visual representations in the FeNO web tool for each of the 3 pieces of information to enter<br><br>Use red, amber and green to indicate well-controlled, possible concerns or poorly controlled for each piece of information | Environmental Context and Resources | Physical opportunity | Environmental restructuring | 12.1 Restructuring physical environment |
| **HCP: Carry out actions recommended by web tool to inform more personalised management of asthma, which may include changes in medication** | | | | | | |
| *FeNO web tool recommendations need to be linked with current local prescribing (EC)* | FeNO web tool | Ensure recommendations of FeNO web tool are in line with prescribing guidelines by liaising with expert stakeholders<br><br>Allow download of FeNO web tool recommendations, to facilitate linking with prescription decisions | Environmental Context and Resources | Physical opportunity | Environmental restructuring | 12.1 Restructuring physical environment |

| **Barriers / *facilitators* to target behaviours** | **Intervention Component/s** | **Intervention ingredient** | **Theoretical Domains Framework (TDF)** | **Target construct (BCW)** | **Intervention function (BCW)** | **BCT (using 93 BCT taxonomy v1)** |
|---|---|---|---|---|---|---|
| *FeNO web tool recommendations need to be simple (EC)*<br><br>FeNO web tool recommendations are too wordy (EC) | Online training<br><br>FeNO Web Tool | Clear, short accessible recommendations for how to manage patient's asthma | Memory, attention and decision process | Psychological capability | Education | 5.2 Salience of consequences |
| HCPs don't know how to step down medication *(EC)* | Online training<br><br>FeNO web tool | Provide a worked example of a HCP stepping down patient medication during the online training<br><br>Provide specific recommendations about how to step down medications at the time it is recommended | Skills | Psychological capability | Training | 4.1 Instruction on how to perform the behaviour |

| **Barriers / *facilitators* to target behaviours** | **Intervention Component/s** | **Intervention ingredient** | **Theoretical Domains Framework (TDF)** | **Target construct (BCW)** | **Intervention function (BCW)** | **BCT (using 93 BCT taxonomy v1)** |
|---|---|---|---|---|---|---|
| Clinicians who are not confident may have some issues with some of the recommendations *(EC)*<br><br>If patients are on high steroids for years, nurses want to leave them as they are, left unnecessary high dose for a long time (I, Pharmacist 07)<br><br>*You can cut down the number of repeated prescriptions in the long term, so cut costs (I, HCP 0209)* | Online training<br><br>FeNO Web Tool | Include a dedicated page about the rationale for stepping down medication, and evidence that patients are keen to stop taking medication when it doesn't benefit them<br><br>Advise that the HCP review the patient in 6-8 weeks to check their FeNO again, to reassure them that they will keep an eye on it | Beliefs about consequences | Reflective motivation | Persuasion | 5.1 Information about health consequences |

| **Barriers / *facilitators* to target behaviours** | **Intervention Component/s** | **Intervention ingredient** | **Theoretical Domains Framework (TDF)** | **Target construct (BCW)** | **Intervention function (BCW)** | **BCT (using 93 BCT taxonomy v1)** |
|---|---|---|---|---|---|---|
| Some HCPs may not have adequate training *(EC)*<br><br>Basic knowledge of asthma is needed *(EC)* | Online training | Provide information about how FeNO helps inform asthma management<br><br>Intervention is designed to be implemented by asthma nurses | Knowledge | Psychological capability | Education | 5.1 Information about health consequences |
| **HCP: Discuss management plan with patients** | | | | | | |
| Communication used as persuasion during asthma consultations (LR) | Online training: video | Provide examples of HCPs discussing management plan with patients and motivating them to follow recommendations by explaining the rationale | Skills | Psychological capability | Training | 4.1 Instruction on how to perform the behaviour |
| **All target behaviours** | | | | | | |
| *Training needs to be easy to follow (EC)* | Online training | Training delivered online in short accessible click-through pages | Skills | Psychological capability | Training | 4.1 Instruction on how to perform the behaviour |

| **Barriers / *facilitators* to target behaviours** | **Intervention Component/s** | **Intervention ingredient** | **Theoretical Domains Framework (TDF)** | **Target construct (BCW)** | **Intervention function (BCW)** | **BCT (using 93 BCT taxonomy v1)** |
|---|---|---|---|---|---|---|
| *Training needs to increase motivation and confidence (EC)* | Online training | Include videos to model taking a FeNO test and interpreting the findings with a patient, and suggest HCP practises using the FeNO analyser | Beliefs about capabilities | Reflective motivation | Education | 6.1 Demonstration of the behaviour |

| | | | | | | | |
|---|---|---|---|---|---|---|---|
| Time constraints (LR)<br><br>Consultation time is limited *(EC)* | FeNO web tool<br><br><br><br>Patient leaflet<br><br><br><br><br>FeNO handout | Ensure FeNO web tool is efficient to use within an asthma review<br><br>Provide patient leaflet in advance of review so that patient knows what to expect<br><br>Provide a short handout that HCPs and patients can use during the review | Environmental Context and Resources<br><br><br><br><br><br><br><br>Memory, attention and decision processes | Physical opportunity<br><br><br><br><br><br><br><br><br>Psychological capability | Environmental restructuring<br><br><br><br><br><br><br><br><br>Training | 12.1 Restructuring physical environment<br><br><br><br><br><br><br>7.1 Prompts/cues | |
| *Nurse education* (LR) | Online training | Ensure that all HCPs undertaking asthma reviews receive training in advance | Skills | Psychological capability | Training | 4.1 Instruction on how to perform the behaviour | |
| *Patient leaflets* (LR) | Patient leaflet | Include a patient leaflet to explain about FeNO testing in advance of asthma review | Knowledge<br><br><br>Beliefs about consequences | Psychological capability<br><br>Reflective motivation | Education | 5.1 Information about health consequences<br><br>6.1 Demonstration of the behaviour | |

EC = Expert consensus (consultation with stakeholders and clinical research team)
LR = Scoping literature review
I = Interview to inform intervention planning
