# Supplementary material for: Optimising stakeholder engagement during intervention planning and development using the Person-Based Approach: the example of an online FeNO-guided asthma management intervention in primary care
Source: NPJ Prim Care Respir Med. 2025 Jul 25;35:33. doi: 10.1038/s41533-025-00435-9 (PMC12297380; doi:10.1038/s41533-025-00435-9)
Supplement: Supplementary file 2 — S2 [file 41533_2025_435_MOESM2_ESM.pdf]

## The Problem

**Sub-optimal management of asthma in primary care**

- No or limited access to FeNO analyser
- Lack of knowledge of how to complete FeNO breath test
- Lack of awareness of how FeNO can inform care
- Uncertainty about how to interpret FeNO readings and using them to discuss management decisions with patients
- Sub-optimal patient adherence to asthma medication and related behaviours

## Intervention components

**FeNO analyser**

**FeNO web-tool (use FeNO measurement in conjunction to other indicators: ACT score, number of exacerbations in the last year)**

**Web-based training with clinical scenarios**

**FeNO handout and discussion with HCPS**

**Patient information leaflet on what FeNO is and how FeNO measurement can inform management**

**FeNO test as part of review**

**FeNO web tool recommendations**

## Behavioural mechanisms

**Physical opportunity:** Environmental context and resources

**Psychological capability:** Knowledge

**Reflective motivation:** Beliefs about consequences

**Physical capability:** Physical skills

**Reflective motivation:** Beliefs about capabilities

**Social opportunity:** Social influences

**Social opportunity:** Social influences

**Psychological capability:** Knowledge

**Reflective motivation:** Beliefs about consequences

**Physical opportunity:** Environmental context and resources

## Purported mediators

**Clinician cognitions**

Belief that FeNO measurement can help them make more informed treatment decision for their patients

Increased confidence in providing more personalised asthma management

**Patient cognitions**

Belief that they will be able to carry on the FeNO test

Belief that FeNO provides (more personalised) asthma management that is beneficial to them

Increased motivation and capability to carry out the behaviours advised as part of the management plan

## Short-term outcomes

**Clinician behaviours**

Explain FeNO measurement and test to patient

Conduct the FeNO test

Use the web tool

Carry out actions recommended by web tool to inform more personalised management of asthma which may include changes in medication*

Discuss management plan with patients

**Patient behaviours**

Carry out the FeNO test during consultation

(Agree to the asthma management plan)

Carry out behaviours (medication adherence) advised in the asthma management plan*

## Long-term outcomes

**Improved management of asthma in primary care**

Reduced frequency of acute asthma exacerbation

Reduced prescribing of inhaled corticosteroids (ICS)

Reduced prescribing of short-acting inhaled beta-agonists (SABA)

Improved asthma control

Improved asthma-related quality of life

* **Clinician adherence:** whether or not clinicians have acted on recommendation from web tool to change medication, assessed through checking medical records. **Patient adherence:** whether or not patient has taken their (new) medication as directed following asthma review, assessed through prescriptions being filled.
