# Supplementary material for: Optimising stakeholder engagement during intervention planning and development using the Person-Based Approach: the example of an online FeNO-guided asthma management intervention in primary care
Source: NPJ Prim Care Respir Med. 2025 Jul 25;35:33. doi: 10.1038/s41533-025-00435-9 (PMC12297380; doi:10.1038/s41533-025-00435-9)
Supplement: Supplementary file 3 — S3 [file 41533_2025_435_MOESM3_ESM.docx]

S3. Think-aloud interviews outcomes

| **Participants** | **Intervention component** | **Key Feedback** | **Key changes** |
| --- | --- | --- | --- |
| 11 HCPs (6 nurses, 4 pharmacists and 2 GPs) | Algorithm | - Very clear, easy to read. - Interesting, useful, good case scenarios - Want to use FeNO test in practice - Liked the idea of the FeNO web tool - • Found session 2 confusing, on how to use algorithm | - Made sessions shorter and simplified some text - Session 1: clarified FeNO test can be used for all people with asthma and included example video on how to conduct FeNO test - Session 2: added content on using FeNO and web tool during consultation, added FAQs, will add video on how to use the web tool |
| 7 patients with asthma | Patient booklet | - Very clear, very interesting, useful information on “controlled doesn’t mean you’re not at risk of asthma attack” - Clear instructions on how to do the test - Want different picture on page 2 - Want to know if if they can blow for 10 seconds even if they are having a bad day - Don’t understand difference between medium and high FeNO - Want to see results | - Page 4: “How do I do a FeNO test”: added information how easy it is to use, normal to try again, give it a go on a bad day - Page 5: normal vs higher FeNO, added results, moved the paragraph on personalized treatment at the end |
